# Multicondition expression profiling reveals limitations of canonical housekeeping genes

**DOI:** 10.1186/s12864-026-12563-8

**Published:** 2026-01-27

**Authors:** Elias Projahn, Michael Walter, Georg Fuellen, Steffen Möller

**Affiliations:** 1 Institute for Biostatistics and Informatics in Medicine and Ageing Research, Rostock University Medical Center, Rostock, Germany; 2 Institute for Clinical Chemistry and Laboratory Medicine, Rostock University Medical Center, Rostock, Germany

**Keywords:** Housekeeping genes, Ubiquitous genes, Reference genes, Expression data, RNA-seq, qPCR

## Abstract

Housekeeping genes are commonly used as reference genes for analyzing expression data. We introduce an algorithm that scores and ranks genes based on their expression ubiquity using three criteria: the proportion of samples with high expression, overall expression levels, and expression invariance. The resulting continuous and configurable measure of gene ubiquity overcomes the limitations of current categorical classifications.

Using this measure, we systematically evaluate how genes traditionally assumed to be ubiquitously expressed (so-called stable housekeeping genes) respond to drugs. By integrating expression data from the Genotype-Tissue Expression portal (physiological conditions) and the Connectivity Map (drug effects), we identify subsets of genes that maintain strong invariance under chemical stimuli, rendering them particularly suitable as reference genes. At the same time, we show that many seemingly ubiquitous genes are strongly influenced by commonly used drugs. Our findings open up new conceptual perspectives on drug mechanisms and cellular regulatory responses and they call into question the reliability of several classic housekeeping genes.

To facilitate broader application of these results, we present an interactive web interface (https://ubigen.uni-rostock.de*)* that allows researchers to explore gene rankings, adjust scoring criteria and analyze specific gene sets. This platform facilitates the selection of robust, ubiquitously expressed reference genes for experimental applications and deepens our understanding of transcriptional regulation under physiological and chemically perturbed conditions.

**Supplementary Information:**

The online version contains supplementary material available at 10.1186/s12864-026-12563-8.

## Introduction

The term “housekeeping gene” traditionally refers to genes required for basic cellular functions such as maintaining structural integrity, metabolism, or protein synthesis. To fulfill these fundamental roles, such genes are expected to be expressed at relatively constant levels across most tissues and under different conditions [1]. This presumed stability, in addition to their involvement in core cellular processes, has made housekeeping genes central to gene expression research. Consequently, they are widely used as internal controls (or reference points) in expression studies to normalize data and to render expression levels comparable across samples or experiments. Techniques that rely on such invariant reference points include real-time quantitative polymerase chain reaction (qPCR) [2], western blotting [3] and high-throughput RNA sequencing (RNA-seq) [4].

When approaching housekeeping genes from the perspective of data analysis – specifically for the purpose of data normalization – their original biological definition becomes less important. Instead, the focus shifts toward their expression patterns alone. To reflect this, we use the terms “ubiquitously expressed genes” or simply “ubiquitous genes” rather than “housekeeping genes”. This terminology is in line with previous large-scale expression studies aimed at identifying stable reference genes [5–7]. We define ubiquitous genes as genes that are expressed across most tissues and cell types under a wide range of conditions and with relatively stable expression levels.

With the advent of extensive expression resources such as the Genotype-Tissue Expression project (GTEx) and the Human Protein Atlas (HPA), numerous efforts have been made to identify ubiquitous genes [1, 5–11]. These initiatives employ different methods for filtering or scoring genes, often using expression thresholds to categorically decide whether a gene is expressed in a given sample [5–7, 9]. Other studies incorporate additional criteria that account for the variability of expression across samples [1, 11]. When multiple criteria are considered, they may be combined conjunctively [11], or through arithmetic scoring [10]. Yet, a comprehensive and systematic approach that supports multiple criteria simultaneously remains lacking.

The first objective of this work is therefore to combine some of the previously described criteria into a single score that produces a continuous ranking of genes. This ranking enables both qualitative and quantitative comparisons-an advantage over earlier categorical approaches. There is no need for ubiquity to be constrained to categorical decisions. Continuous scoring allows for a more nuanced and objective selection of reference genes, which we view as a key strength of our approach. Moreover, since the exact definition of gene ubiquity is still debated, the scoring algorithm is designed to be configurable and adaptable, supporting future developments and allowing researchers to emphasize different aspects of ubiquity according to their needs.

Understanding how a single gene or gene set performs within the ubiquity ranking can provide valuable physiological insights. For example, when analyzing expression data, it is informative to distinguish genes that are highly expressed despite being normally non-ubiquitous, or conversely, genes that show unexpectedly low expression despite being typically ubiquitous. Such information is of potential interest for interpreting and contextualizing gene expression studies. Therefore our aim was to make our results easily accessible by providing interactive visualizations and a graphical user interface to customize scoring criteria and explore genes of interest.

In a landmark 2006 study, Lamb and coworkers introduced the Connectivity Map (CMap), which catalogs the transcriptional effects of FDA approved drugs on various cell lines, using Affymetrix GeneChips [12]. Many compounds were tested across different cell lines and different concentrations. Despite the limitations of in-vitro cancer cell models, the CMap offers a valuable overview of how small molecules influence the transcriptome. Building on this resource, we also ask whether genes identified as ubiquitous in large expression datasets such as GTEx or HPA remain ubiquitous under chemical perturbations as represented in CMap. Genes that maintain stable expression across human tissues, physiological conditions, and even under the influence of a variety of drugs are especially strong candidates for use as reference or control genes. Conversely, genes that appear ubiquitous under physiological conditions but vary substantially under chemical influence may provide insight into drug mechanisms, cellular stress responses, or dose-dependent toxicities. Surprisingly, we find that a considerable proportion of genes traditionally referred to as housekeeping genes are strongly affected by many compounds.

## Results

### The ubigen score quantifies expression ubiquity

To capture how ubiquitously a gene is expressed, we integrated multiple criteria into a single composite score. These criteria can be grouped into three classes. The first and main class refers to the expression breadth across samples, considering the proportion of samples in which a gene is “highly” expressed. The definition of “highly expressed” is configurable in our interactive user interface, as different analyses may require different thresholds. Our default threshold is the 95th percentile expression value, a choice that increases specificity. Thus, this subscore represents the fraction of samples in which a gene’s expression level meets or exceeds the sample-specific 95th percentile. Users may alternatively choose cut-offs based on the median expression level per sample or simply on whether the gene is detectable at all. The remaining two classes capture the overall expression level and expression variability. By default, we use the median expression level across samples to quantify magnitude, and the quartile-based coefficient of variation (interquartile range divided by median) to quantify variability. Table 1 lists the different criteria, their underlying rationale, and their default weights in the overall Ubigen score.

**Table 1:** Criteria for scoring gene ubiquity

Class	Criterion	Description	Weight
Expression across samples	Above 95th percentile (default)	Fraction of samples in which a gene is expressed above a specified threshold. The threshold is calculated separately for each sample to ensure independence from overall expression levels. An “above zero” threshold corresponds to the canonical definition of ubiquitous genes – those expressed in all tissues and under all conditions. More stringent thresholds yield increasingly specific selections.	+ 50%
	Above median		
	Above zero		
Expression level	Median (default)	The expression level across all samples. Ubiquitous genes are expected to be robustly expressed at a consistent and biologically meaningful level.	+ 25%
	Mean		
Expression variation	Quartile-based coefficient of variation (default)	Quantification of the variability of gene expression across samples. By default, it is negatively weighted to emphasize expression invariance. A truly ubiquitous gene should exhibit relatively stable expression regardless of tissue type or experimental condition.	−25%
	Interquartile range		
	Coefficient of variation		
	Standard deviation		

This table summarizes the criteria used to score genes for ubiquity. The criteria are organized into three classes, from which one criterion per class is selected to compute a combined score. For each class, the table also describes the underlying rationale and lists the default criterion and its associated weight, which together define the gene’s Ubigen score

For each of the three classes, one criterion is selected for scoring all genes, yielding three subscores. Each subscore is scaled to the range [0.0, 1.0] using min-max normalization so that their dimensions are comparable. The three normalized subscores are then combined into a single value by computing a weighted average. The resulting composite score is again min-max normalized to [0.0, 1.0] because different combinations of weights would otherwise produce scores with non-comparable ranges. Together, these steps allow users to freely configure and weight the different criteria, tailoring the score to their preferred concept of ubiquity. Table 1 shows the default criteria and weights that implement our default definition of ubiquity. In the default setup “Expression across samples” receives the highest emphasis, reflecting its strong alignment with the classical notion that housekeeping genes are expressed under all conditions. The default criteria for overall expression level (median) and expression variation (quartile-based coefficient of variation [QCV]) were chosen to reduce redundancy, enabling independent weighting. This is achieved by normalizing the interquartile range based on the median resulting in the QCV as a robust measure of variation. We refer to the score produced by this default configuration and described in Table 1a gene’s Ubigen score.

**Fig. 1:**
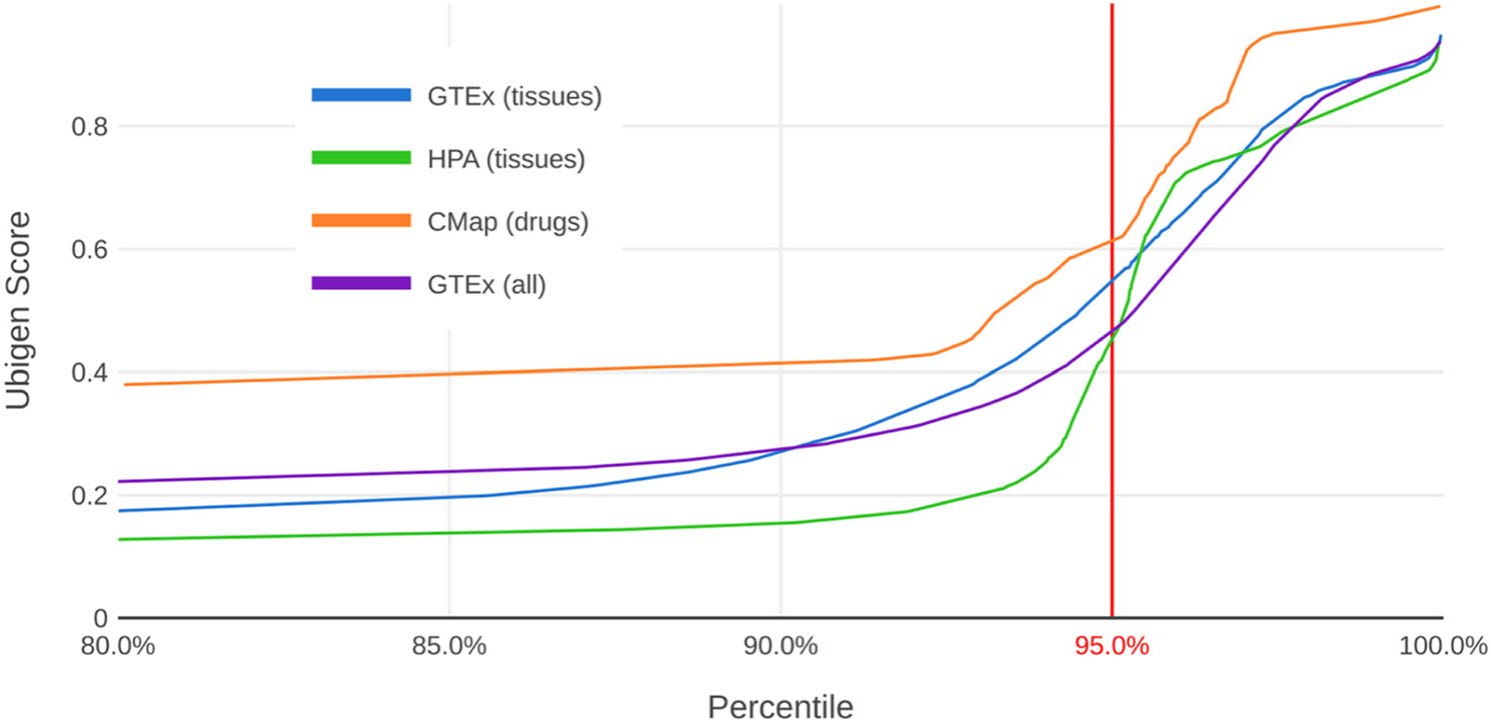
Distribution of Ubigen scores for different datasets. The plot shows the distribution of Ubigen scores derived from different datasets, restricted to the top 20% of each ranking (the X-axis starts at the 80th percentile). “GTEx (all)” computes scores directly from all GTEx samples encompassing diverse tissues and conditions. In contrast, “GTEx (tissues)” and “HPA (tissues)” rely on tissue-aggregated expression values, using a single representative value per tissue. “CMap” is based on expression data measured under the influence of different drugs and uses summary statistics computed per drug

While the selection of default weights can be seen as an arbitrary choice, the selected configuration satisfied three desirable properties: First, with the 95th percentile located at a score of approximately 0.5, the resulting distribution has a high selectivity (Fig. 1). This is an advantage for the targeted selection of ubiquitous genes. Second, the weights reflect a balanced combination of the two core aims: seeking candidate genes based on ubiquity (expression across samples) and confirming that those candidates are robustly expressed (sufficient expression level and low variation). Third, the results are stable with respect to modest changes in the weights. To assess this stability, we performed a sensitivity analysis in which all weights were varied by ± 20% (expression across samples [40%, 60%], expression level [20%, 30%], expression variation [−30%, −20%]) and evaluated 1,331 equally distributed combinations within these ranges. The overlap across the top 5% of all resulting rankings is 99.8% for the “GTEx (all)” dataset. When looking at the intersection of “GTEx (all)” and “CMap”, i.e., sector 11 of Fig. 3, the overlap remained high at 98.7%.

### Different input datasets produce similar scores

We compared different publicly available datasets to assess how robustly the Ubigen score generalizes across different sources of gene expression data. The following four prominent datasets were used as input data to the algorithm: “GTEx (all)” is a custom dataset based on re-normalized read counts from the GTEx project without tissue-level aggregation; “GTEx (tissues)” uses the tissue-aggregated expression levels as published by the GTEx project; “HPA (tissues)” uses similar data from the Human Protein Atlas; “CMap” is the dataset previously described in Struckmann et al. 2021, that provides summary statistics quantifying gene responses to chemical perturbations derived from CMap [13].

Across all four datasets, the resulting score distribution exhibits a similar shape (Fig. 1). There are a large number of genes (about 90%) with low scores. At the 95th percentile or shortly before, the slope of the distribution increases. We therefore decided to put the threshold for ubiquity (as a quality, not a quantity) at the 95th percentile, which means that we consider 5% of genes to be ubiquitous genes. We can give an absolute number only per dataset because the datasets contain different numbers of genes (number of ubiquitous genes/total number of genes: 2,762/55,242 for “GTEx (all)”; 2,210/44,219 for “GTEx (tissues)”; 980/19,616 for “HPA (tissues)”; 619/12,382 for “CMap”).

Among the datasets based on physiological conditions, the ranking based on “GTEx (all)” results in the smoothest distribution (Fig. 1). This results from the much larger number of data points after eliminating the intermediate step of aggregating data per tissue (17,382 samples for “GTEx (all)” vs. 54 tissues for “GTEx (tissues)”/253 tissues for “HPA (tissues)”). The “GTEx (all)” dataset also has the lowest correlation to the “CMap” dataset (ρ = 0.4475; ρ = 0.5261 for “HPA (tissues)”; ρ = 0.5334 for “GTEx (tissues)”; Spearman’s rank correlation coefficient), making a comparison of these two datasets more informative. As expected, scores for the three datasets without drug effects are highly correlated with one another (Supplementary Table 1). Based on these analyses, we decided to use the scores based on “GTEx (all)” for all subsequent calculations and for the comparison to the “CMap” score. There are three reasons for this decision: the much larger sample size, the smoother and more granular distribution and the low correlation with the “CMap” score.

### Sliding gene set enrichment analysis shows more annotations for higher scores

**Fig. 2:**
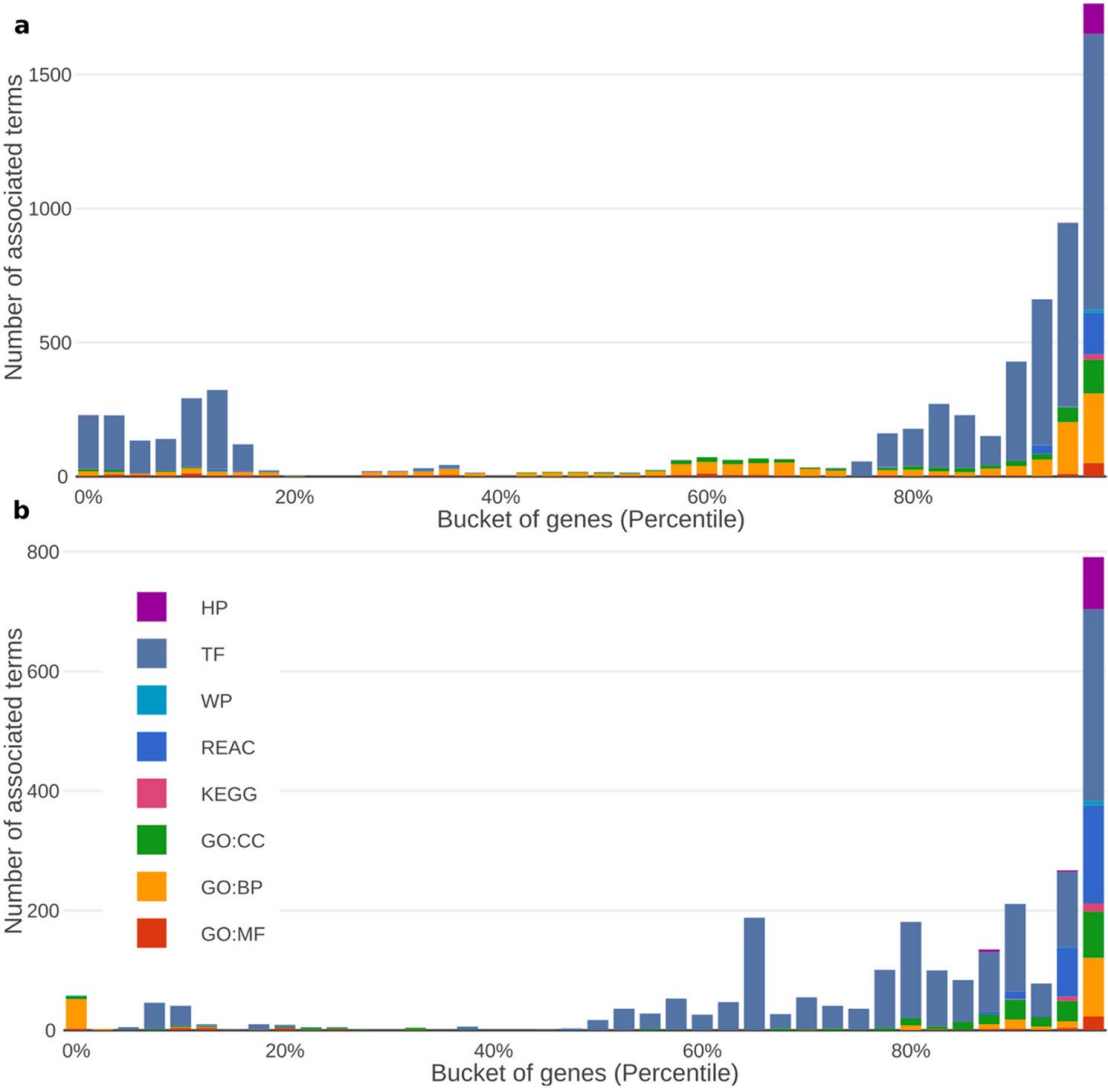
Sliding gene set enrichment analysis along the ranking. This figure shows a sliding gene set enrichment analysis along the ranking of human genes based on Ubigen scores from the “GTEx (all)” (**a**) and “CMap” (**b**) datasets. For each bucket containing 2.5% of all analyzed genes, a gene set enrichment analysis was performed using g:Profiler. In both datasets, the number of significant associations with terms from diverse annotation sources increases markedly toward the top of the ranking. The color coding corresponds to the human phenotype database (HP), transcription factors (TF), WikiPathways (WP), Reactome (REACT), KEGG and the three ontologies of the Gene Ontology consortium: cellular components (GO:CC), biological processes (GO:BP), and molecular function (GO:MF)

We previously applied a sliding gene set enrichment analysis to assess the biological relevance of gene rankings [14]. By dividing the data into equally sized buckets, each containing 2.5% of the genes, we can visualize how certain sources of annotations are highlighted along the ranking (Fig. 2). Both the “GTEx (all)” (Fig. 2a) and “CMap” dataset (Fig. 2b) result in rankings where terms from all sources included in g: Profiler are enriched at the top. In both cases, the top 2.5% of genes have the maximum number of associations for all sources. This pattern underscores the biological significance of the ranking: genes with many annotations are typically key players in a wide range of biological processes and regulatory mechanisms. For example, the top 2.5% of genes in the “GTEx (all)” ranking are strongly enriched for genes involved in fundamental pathways such as cytoplasmic translation, aerobic respiration, ribosome biogenesis, and regulation of RNA splicing (Supplementary Table 2).

### About 3% of analyzed genes are potentially suitable reference genes for experiments

**Fig. 3:**
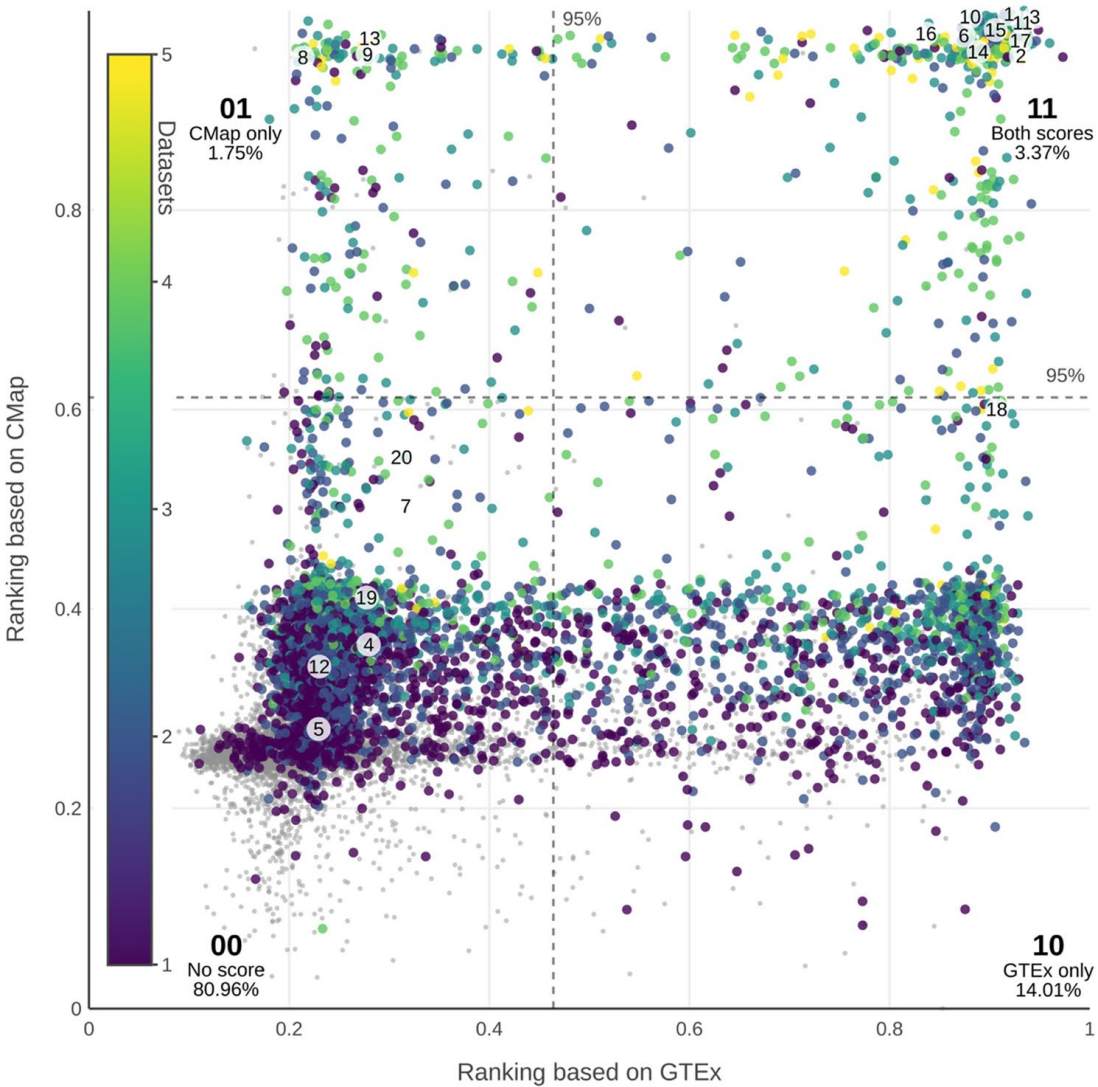
Ubigen scores for genes previously identified as ubiquitously expressed across large expression datasets. The plot shows Ubigen scores based on “CMap” (Y-axis) in relation to scores based on “GTEx (all)” (X-axis) for all genes common to both datasets (N = 12,323). The two dashed lines indicate the 95th percentile of the scores for each dataset. Based on this, the four sectors are named (00, 01, 10, 11) and the percentages of genes in each sector are given. Twenty genes frequently cited in the literature as common housekeeping and reference genes (listed in Table 2) are highlighted and numerically annotated. The color coding reflects the number of previous studies on ubiquitous genes in which each gene is identified as ubiquitous, while genes absent from all referenced datasets are shown as gray markers in the background

We next sought to estimate the proportion of genes that might serve as robust reference genes. To do this, we compared the “GTEx (all)” score (representing physiological conditions) with the “CMap” score (representing drug-induced effects). Among the 12,323 genes shared by both datasets, we examined how genes classified as ubiquitous or non-ubiquitous in each dataset overlap. Figure 3 shows the combined scores and introduces four sectors that represent all possible combinations of ubiquity: As previously described, the top 5% of genes in each dataset are considered ubiquitous. Thus, a gene can fall into one of four categories: ubiquitous in both datasets (sector 11), ubiquitous in only “GTEx (all)” (sector 10), ubiquitous in only “CMap” (sector 01) or non-ubiquitous in both (sector 00).

The combined analysis reveals four clearly separated clusters corresponding to these sectors. As one may expect, most genes fall into sector 00 (n_00_ = 9977, 80.96%), where neither score indicates strong ubiquity. Genes considered ubiquitous based on one dataset alone are now split into two groups: a substantially larger group in sector 10 (n_10_ = 1727, 14.01%), representing genes ubiquitous under physiological conditions only, and a small group in sector 01 (n_01_ = 216, 1.75%), representing genes ubiquitous only under drug-induced conditions. Genes that are ubiquitous in both datasets represent a small but biologically meaningful set (n_11_ = 403, 3.27%). These genes seem suitable as reference genes both under physiological conditions and under the influence of medication.

### Only 50% of commonly used reference genes are ubiquitous according to our score

We were interested in comparing our approach to previously published datasets of ubiquitous genes and classical housekeeping genes. We used data from five widely cited studies based on large-scale expression analyses: Warrington et al. 2000 (ref [8])., Zhu et al. 2008 (ref [9])., Eisenberg and Levanon 2013 (ref [11])., Chang et al. 2011 (ref [10]). and Joshi et al. 2022 (ref [1]). Together, these datasets encompass a total of 6,149 genes, with 4,832 genes common to both the “GTEx (all)” and “CMap” datasets (Fig. 3). These datasets differ significantly from each other, suggesting a potential for refinement: A Venn diagram showing all intersections between the datasets (Supplementary Fig. 1) reveals only 88 genes present in all five datasets (Supplementary Table 3).

Figure 3 visualizes the distribution of these genes in our results with color coding. Relative to the total number of genes in sector 11, each ubiquitous gene dataset is highly overrepresented in that sector: 51.12% from Warrington (2000), 83.37% from Zhu (2008), 52.61% from Eisenberg (2013), 89.33% from Chang (2011), 43.18% from Joshi (2022) (Supplementary Fig. 2). However, only a minority of genes from each dataset fall into sector 11: 44.99% for Warrington (2000), 17.99% for Zhu (2008), 7.41% for Eisenberg (2013), 17.73% for Chang (2011), 6.53% for Joshi (2022). This indicates that, while genes from sector 11 are a strong predictor for inclusion in these datasets, the reverse is not the case: With the exception of Warrington (2000), where 55.01% of genes also lie outside sector 11, none of the datasets reliably predict genes that belong to this sector.

Interestingly, when looking at the intersection of these studies and those that explicitly focus on housekeeping genes, a larger proportion of the genes are found in sector 11. Of the 88 genes common to all five datasets, 54.55% are located in sector 11. Additionally, most genes frequently cited as reference genes in literature reviews on housekeeping genes [15–17] also fall into sector 11. These genes are highlighted in Fig. 3, with detailed results listed in Table 2.

**Table 2:**
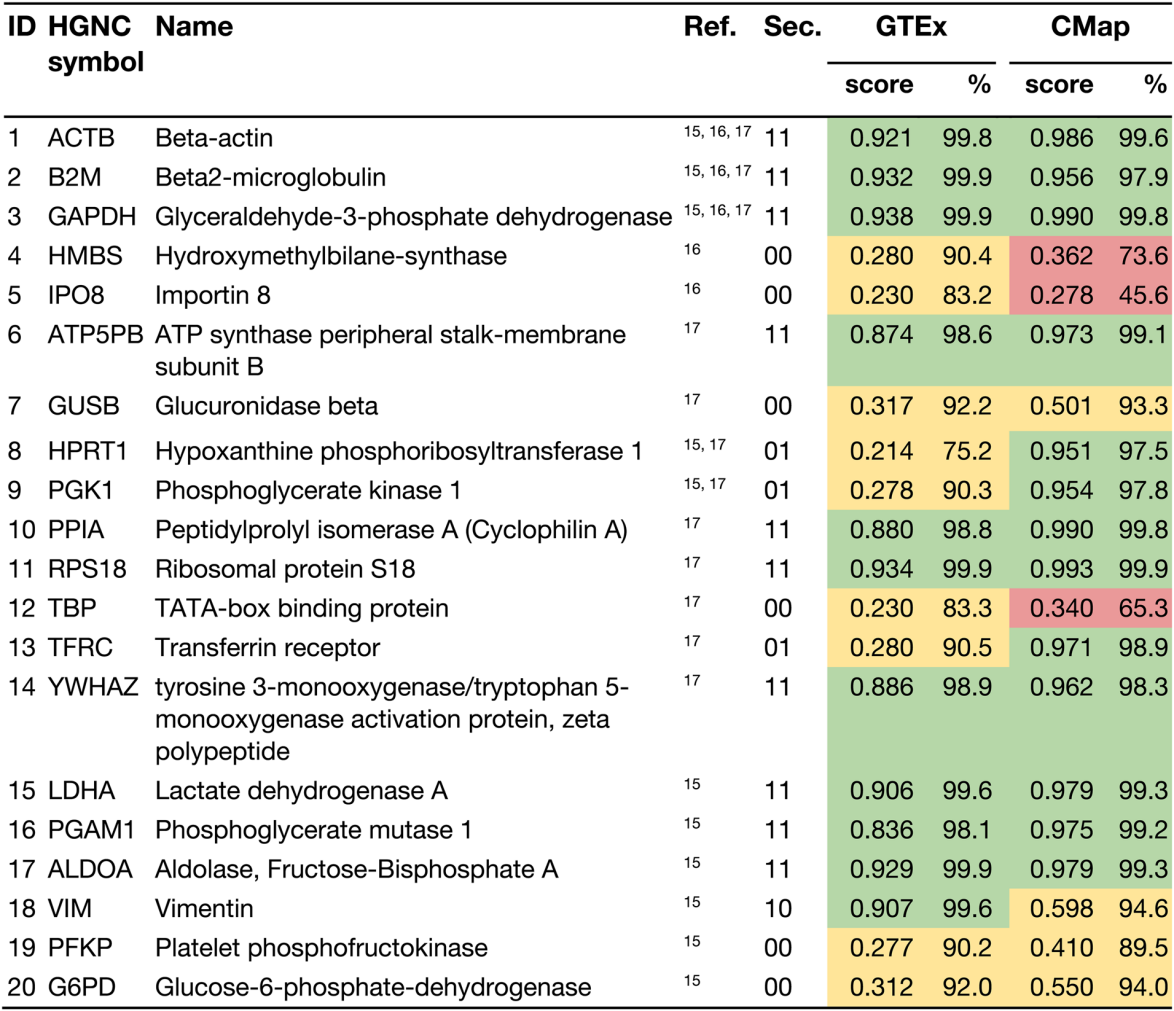
Ubigen scores for commonly used housekeeping genes

This table shows the Ubigen scores for housekeeping genes commonly used as reference genes, as reported in the literature. The gene identifiers in the first column correspond to those in Figure 3. The “Sec.” column indicates the sectors based on Figure 3. The displayed values are as follows (from left to right): Ubigen score based on data from the “GTEx (all)“ dataset; rank (percentile in %) within the distribution of scores from the “GTEx (all)” dataset; Ubigen score based on data from the CMap dataset; rank (percentile in %) within the distribution of scores from the “CMap” dataset. Red: Scores below the 75th percentile; Yellow: Scores between the 75th and 95th percentiles; Green: Scores above the 95th percentile. The GTEx (all) Score reflects gene stability under diverse physiological conditions, while the CMap score accounts for the effects of drug treatment

Our findings suggest that many commonly used housekeeping genes exhibit stable expression patterns both under basal conditions and in response to drug treatments, making them suitable as reference genes. Notable examples include *GAPDH* (mean score of “GTEx (all)” and “CMap” = 0.9640), *RPS18* (0.9635), *ALDOA* (0.9540), *ACTB* (0.9535) and *B2M* (0.9440), as well as *LDHA* (0.9425), *PPIA* (0.9350), *YWHAZ* (0.9240), *ATP5PB* (0.9235) and *PGAM1* (0.9055). However, there are also classical housekeeping genes that, according to our evaluations, are not suitable as reference genes or are only suitable to a limited extent. These include *HMBS*, *IPO8*, *GUSB*, *HPRT1*, *PGK1*, *TBP*, *TFRC*, *VIM*, *PFKP*, and *G6PD*. Some of these genes, such as *HMBS*, *IPO8*, and *TBP*, stood out with especially low “CMap” scores, indicating they are strongly influenced by drug treatments and are therefore unsuitable as stable reference genes.

### Despite the broad impact of drugs, such as antineoplastics, on the expression of ubiquitous genes, a subset of genes remains suitable for use as reference controls

**Fig. 4:**
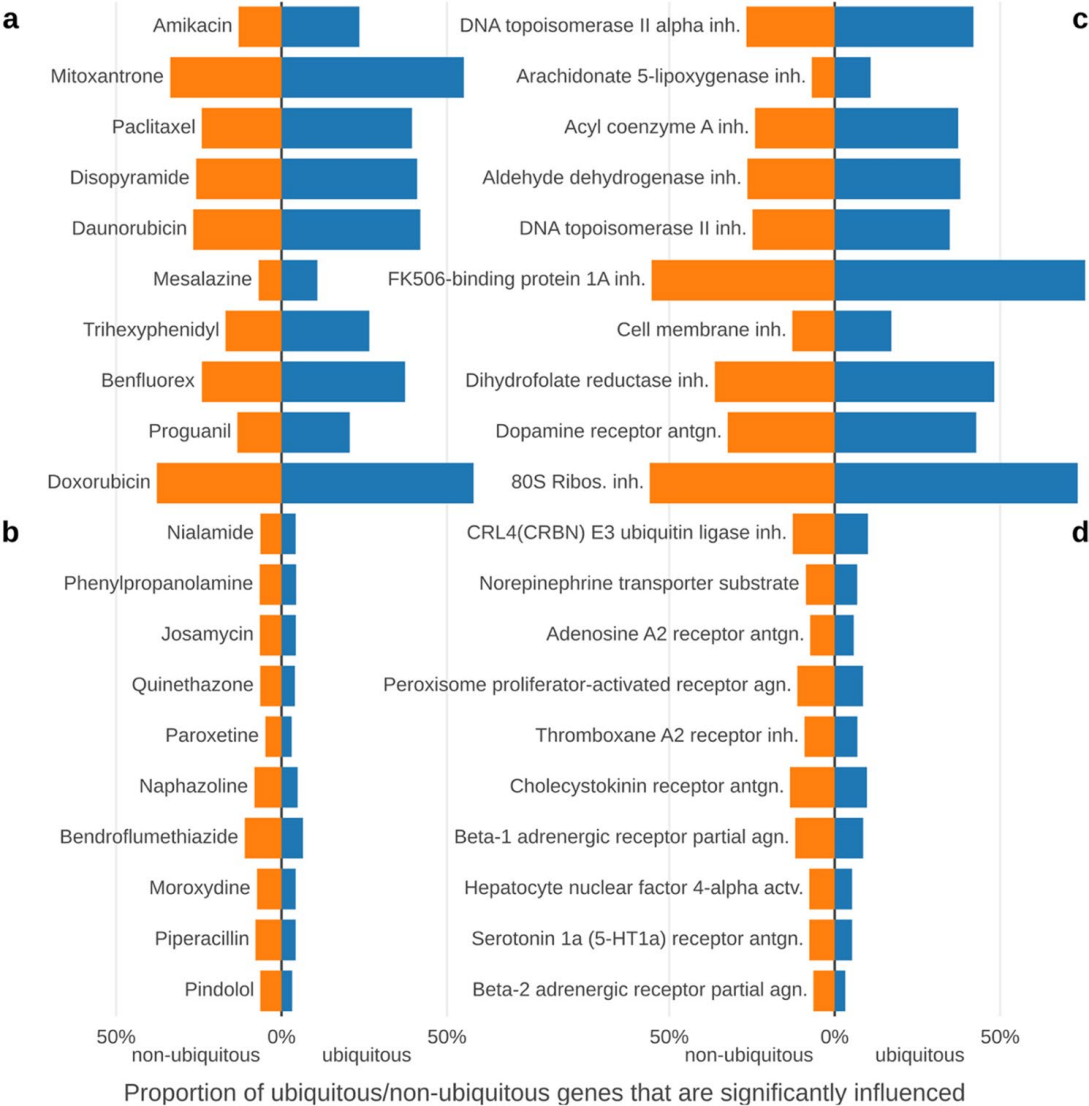
Drug effects on non-ubiquitous and ubiquitous genes. This figure illustrates the proportion of ubiquitous and non-ubiquitous genes (based on “GTEx (all)” and restricted to genes also present in “CMap”) that are significantly influenced by a selected set of drugs. Panels **a** and **b** (left) show results for individual drugs, while panels **c** and **d** (right) present drug groups categorized by mechanism of action. Items displayed in the figure were chosen based on their relative bias toward ubiquitous genes, measured as the ratio of non-ubiquitous to ubiquitous genes. The top ten items exhibit the strongest bias toward ubiquitous genes (**a**, **c**), whereas the bottom ten are biased toward non-ubiquitous genes (**b**, **d**)

The data described above clearly show that drugs can affect genes with high Ubigen scores based on “GTEx (all)”, including genes widely referred to as housekeeping genes. To investigate this further, we asked how a gene that is normally expressed ubiquitously becomes non-ubiquitous (sector 10 genes). We examined this at the level of individual drugs (Fig. 4ab) and mechanisms of action (MOA, Fig. 4cd). By comparing the proportion of ubiquitous genes (Ubigen scores at the 95th percentile and above) significantly influenced by a drug with the proportion of non-ubiquitous genes (Ubigen scores below the 95th percentile), we can assess how strongly a drug or MOA drives genes into sector 10, thereby making them non-ubiquitous due to drug exposure.

Drugs and MOAs with the strongest bias toward affecting ubiquitous genes (Fig. 4ac) also tend to exert broad, global effects on gene expression. In contrast, those biased toward affecting non-ubiquitous genes (Fig. 4bd) generally influence only a small number of genes and appear more specific. Among the drugs and MOAs most biased toward affecting ubiquitous genes are many cytostatics, the anticholinergic trihexyphenidyl, the serotonin agonist benfluorex, and several antibiotics such as amikacin and proguanil. Conversely, drugs and MOAs with the strongest bias toward affecting non-ubiquitous genes include many specialized agents, such as monoamine oxidase inhibitors (e.g., nialamide), the selective serotonin uptake inhibitor paroxetine, the β-sympathomimetic phenylpropanolamine, the β-blocker pindolol, as well as some antibiotics (e.g., josamycin, piperacillin).

A more detailed and quantitative comparison of individual drugs can be obtained by weighting the average Ubigen scores of drug-affected genes by the corresponding log fold change. An interactive visualization of this approach is available online in our web interface (https://ubigen.uni-rostock.de*)* on the “Additional information” page, and a preview is provided in Supplementary Fig. 3. To quantify drug influence, we used absolute log fold-change values, meaning that up- and downregulation are not distinguished.

When comparing overall drug effects across the four sectors, the strongest effects are observed for genes in sector 10 (Supplementary Fig. 4). These genes are ubiquitous under physiological conditions but lose this property under the influence of drugs, indicating that substantial regulatory perturbations are required to affect normally ubiquitous genes. However, genes in sector 11 – genes that remain ubiquitous even under drug exposure – are also significantly impacted. Notably, antineoplastics and immunomodulating agents (WHO ATC code L) induce particularly strong expression changes.

In summary, drugs exert substantial influence both on gene expression levels and on whether a gene remains suitable as a reference gene. Surprisingly, this includes genes classified as ubiquitous in both the “GTEx (all)” and “CMap” rankings – genes that would typically be presumed stable enough to serve as reference genes in expression studies. Understanding this apparent contradiction motivates the analyses presented in the following sections.

### Gene set enrichment analysis reveals differences between the GTEx and CMap score

**Fig. 5:**
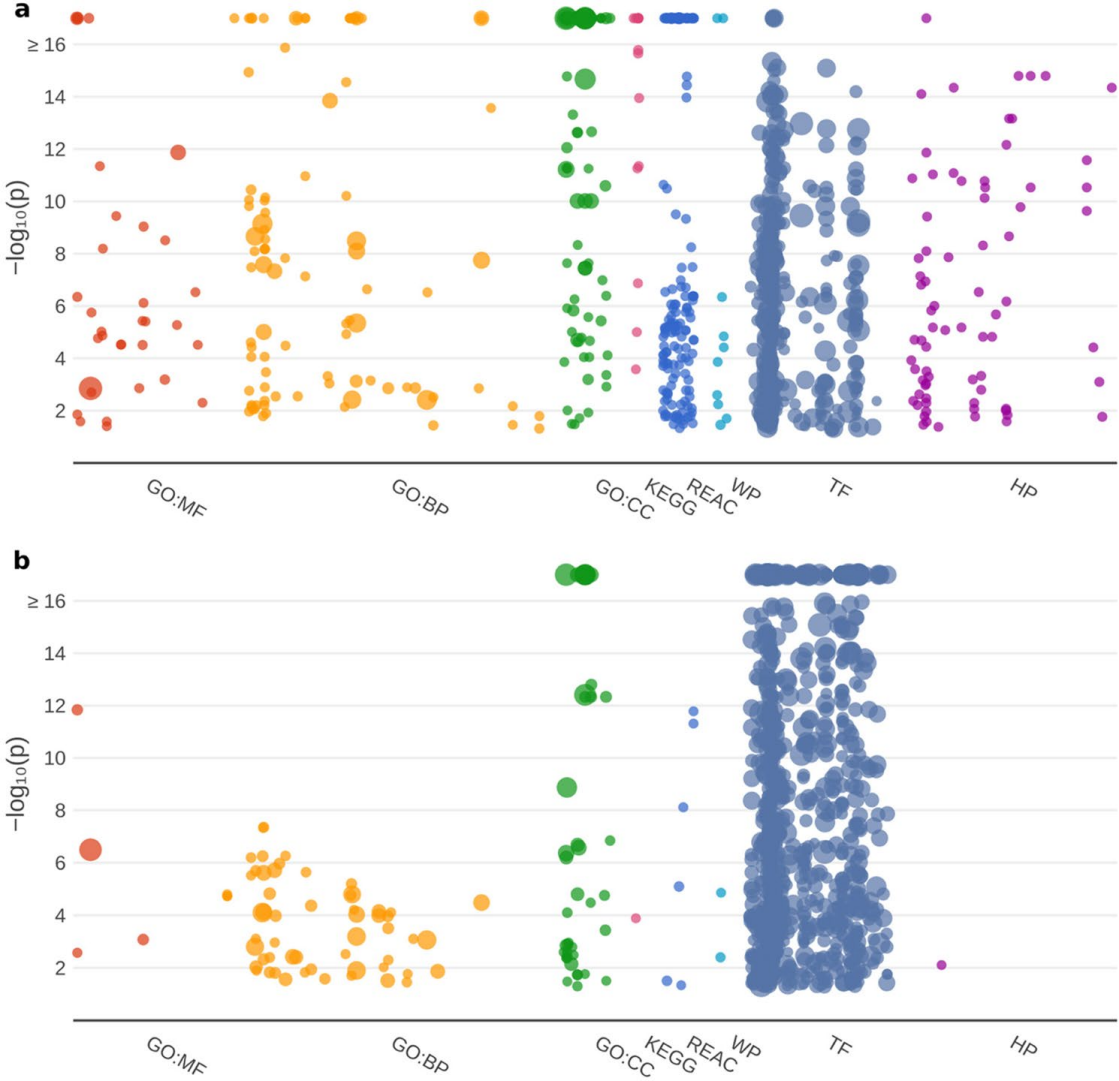
Gene set enrichment analysis for ubiquitous genes. This figure presents the results of gene set enrichment analyses for sector 11 genes (**a**) and sector 10 genes (**b**), as defined in Fig. 3. Both gene sets show numerous significant associations, but clear differences emerge between them. Sector 11 genes – those that remain ubiquitous even under drug influence – exhibit substantially stronger enrichment for Gene Ontology categories, including molecular functions (GO:MF), biological processes (GO:BP) and cellular components (GO:CC). This contrast is even more pronounced for pathway-related annotations from KEGG, REAC, and WP. In contrast, transcription factor binding site (TF) annotations are more enriched in sector 10 genes, which are ubiquitous only in GTEx but not under drug exposure. For visualization, the Y-axis is capped at 16

Consistent with the observation in Fig. 2, genes ranking highly in both the “GTEx (all)” and “CMap” lists are associated with numerous terms from Gene Ontology and other annotation sources. The analyses for Fig. 2 considered each ranking independently. Although one could define a combined score to perform a joint sliding gene set enrichment analysis, more informative patterns emerge from comparing sector 11 (genes ubiquitously expressed across datasets) and sector 10 (genes ubiquitous only in GTEx). To explore these differences, we performed two separate gene set enrichment analyses using g: Profiler [18] and generated the corresponding plots to summarize contributing annotation sources and the significance of detected associations (Fig. 5).

Both sectors show a high number of significant associations. However, when considering the overall number of associations per source and the number of highly significant associations (-log_10_(p) ≥ 16), clear differences emerge. Sector 11 genes – those that remain ubiquitous even under drug influence – display strong enrichment for terms related to molecular functions (GO: MF), biological processes (GO: BP), biochemical pathways (KEGG, REAC, WP), and human phenotype variation (HP). In contrast, transcription factor binding sides (TF) are more prominently enriched among sector 10 genes, which are not ubiquitous under drug influence.

### Interactive user interface

**Fig. 6:**
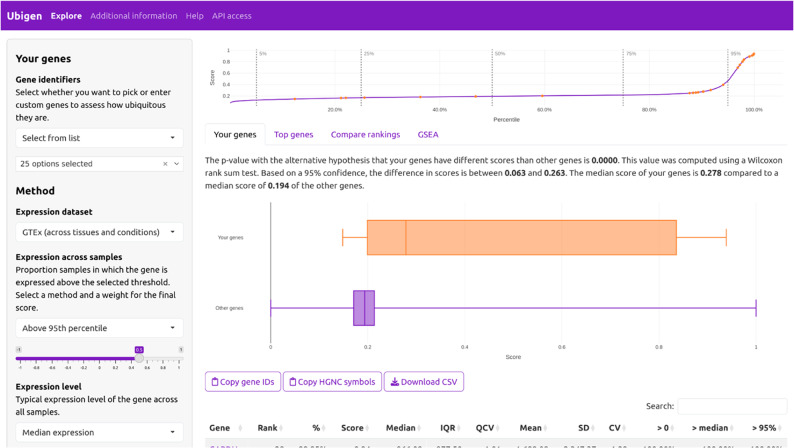
Screenshot of interactive web interface. The example shown uses the default Ubigen ranking to evaluate genes involved in glycolysis according to the KEGG pathways database (KEGG:hsa00010+M00001). The positions of these genes are displayed at the top, and a box plot summarizes their score distribution relative to all genes. The interface supports parameter adjustments and interactive dataset downloads

Determining whether a gene or a set of genes is ubiquitously expressed can be an important question for researchers. To support such analyses, we developed an interactive web interface, available at https://ubigen.uni-rostock.de, which enables users to analyze genes of interest, download data and visualizations, and adjust the scoring method by modifying criteria and their weighting (Fig. 6). As with the comparison between the “GTex (all)” and “CMap” rankings, user-defined configurations and datasets can be compared using a visualization similar to Fig. 3. A help section describing common usage patterns is accessible from the top bar menu, and custom datasets can also be downloaded directly through the interface.

We highlight three use cases that demonstrate how the method can be applied on different levels: (1) the evaluation of specific candidate reference genes, (2) analyzing a gene set of interest, and (3) comparing entire expression datasets.

Ad 1: According to de Jonge et al. (2007), vimentin (*VIM*) is a housekeeping gene commonly used as a reference gene [15]. Using the interactive interface, its Ubigen score in “GTEx (all)” is 0.91 (99.6th percentile). However, the automatically generated ranking-comparison plot (under “Compare rankings”) shows that *VIM* drops below the 95th percentile in the “CMap” dataset (score 0.6, 94.6th percentile), placing it in sector 10 of Fig. 3. This indicates that *VIM* is not a robust reference gene under chemically perturbed conditions.

Ad 2: The interface allows users to submit custom gene lists-such as pathway-defined sets-to assess their ubiquity profiles, visualize their distribution within the ranking, and compare them against background datasets. For example, using the “Sample data: Common reference genes” option, users can immediately evaluate other genes from Table 2. In the “Compare rankings” tab, *RPS18* and *GAPDH* appear in the top-right corner, identifying them as the most stable candidates across both physiological and drug-perturbed conditions.

Ad 3: A third usage scenario is to compare the overall expression behavior of genes within large expression datasets. The comparison between “GTEx (all)” and “CMap” for finding ubiquitous genes is highlighted in this paper. A second practical example is a dataset based 1,244 glioma cases from The Cancer Genome Atlas Program (TCGA) [19, 20]. Selecting the same comparison genes as before and choosing “Compare datasets” and “Glioma (TCGA)” reveals that *ALDOA*, previously placed in sector 11 and considered a reliable reference gene, becomes a strong outlier in glioma. This demonstrates that *ALDOA* should not be used as a reference gene for glioma expression analyses and illustrates how the method might be useful for selecting suitable reference genes in cancer research.

### External validation using expression data from glioma

This analysis is based on thousands of samples across 54 different tissues and 1,206 drugs. Assessing individual gene-drug combinations would yield only incidental information, making direct laboratory validation not feasible. As an alternative, we approximated large-scale experimental validation by using the “Glioma (TCGA)” dataset as an independent expression source. This allowed us to assess whether genes from sector 11 of Fig. 3 are suitable reference genes in an independent realistic application.

The selection of reference genes for cancer research is challenging because tumors often exhibit atypical expression patterns. In glioma, 89.8% of genes from sector 11 remain ubiquitously expressed, indicating that they are strong candidates for reference genes. For comparison, if one were to choose genes from both sectors 10 and 11 without distinction, only 77.6% would meet the criteria, demonstrating a clear improvement over the status quo. When focusing on the commonly used reference genes listed in Table 2, nine of ten sector 11 genes prove suitable in glioma (*ACTB*, *B2M*, *GAPDH*, *ATP5B*, *PPIA*, *RPS18*, *YWHAZ*, *LDHA*, *PGAM1*). In contrast, among the ten other commonly used reference genes that do not fall into sector 11, only one (*VIM*) would be appropriate in this context.

## Discussion

The terms “ubiquitous genes”, “housekeeping genes” and “reference genes” describe related but conceptually distinct properties. “Ubiquitous” refers to a purely quantitative measure of expression breadth, “housekeeping” implies a biological role essential for basic cellular function, and “reference” denotes practical suitability for normalization in experimental applications. The Ubigen score introduced here provides a continuous and configurable measure of gene ubiquity. This approach improves the identification of suitable reference genes for in vitro experiments and also offers new insights into cellular biology under both physiological and drug-induced conditions.

### Identification of reference genes

Unlike categorical classifications, the Ubigen scoring system allows for nuanced, quantitative comparisons across datasets. We leveraged data from the Genotype-Tissue Expression (GTEx) Portal, the most comprehensive public resource for tissue and cell-specific gene expression and regulation across individuals, developmental stages, and species. GTEx includes samples spanning diverse age groups and captures aspects of metabolic variability and dysfunction [21]. In addition, we incorporated data from the Connectivity Map (CMap), which comprises over 1.5 million gene expression profiles derived from approximately 5,000 small-molecule compounds and 3,000 genetic perturbations [12]. The combined use of GTEx and CMap improved the identification of reliable reference genes (sector 11) by explicitly accounting for resilience to external perturbations and by increasing confidence in the classification of ubiquitous genes.

Among commonly used reference genes, *GAPDH* ranked highest according to the Ubigen score (Table 2). Nevertheless, several robust alternatives exist for cases in which *GAPDH* is itself a research target or when experimental conditions compromise its stability as a reference gene. *GAPDH* belongs to a gene cluster with the highest Ubigen scores across datasets, along with eleven genes exhibiting even higher scores within the same cluster (Fig. 3, Supplementary Table 4). These include *MT-ND5*, which encodes a component of respiratory complex I; *FTL*, a subunit of ferritin involved in intracellular iron storage; and *TMSB4X* and *TMSB10*, which play roles in actin remodeling [22]. Ribosomal components such as *RPS27*,* RPL8*,* RPLP1*,* RPL13*,* RPL13A*,* RPL7A* and *RPS6* are also represented in this high-scoring group.

Notably, several classical housekeeping genes fall short of our ubiquity criteria, suggesting that their widespread use as reference genes should be reconsidered. Genes such as *TBP*,* HMBS* and *IPO8* show pronounced drug-dependent expression changes. Furthermore, other commonly cited reference genes, including *GUSB*,* HPRT1*,* PGK1*,* TFRC*,* VIM*,* PFKP* and G6PD, exhibit comparatively low values in the “GTEx (all)” score, indicating limited suitability as universal reference genes under diverse experimental conditions.

### Identification of drug targets and mechanisms

Ubigen scoring is not only informative for quantifying gene ubiquity but also reveals biologically meaningful patterns relevant to drug action. Drug-induced expression changes in genes that are normally stably expressed can substantially affect the interpretation of experimental results. A reasonable initial assumption was that most drugs act relatively specifically and would therefore preferentially target non-ubiquitous genes (sector 00), which exhibit greater physiological variability and more restricted expression. However, our results challenge this view or even suggest the opposite. We found that the largest effect sizes are observed for genes that are ubiquitous in “GTEx (all)” but not across all conditions (sector 10). We interpret this as insight into the primary mode of drug action: many drugs exert their effects by perturbating genes essential for fundamental cellular processes, thereby altering physiological homeostasis rather than acting exclusively on condition-specific targets.

The distinction between sectors 11 (general ubiquity) and 10 is particularly informative. Genes in sector 11 generally remain stable even under drug exposure. Although drug-associated effect sizes in this sector are smaller than those observed in sector 10, they are still larger than those in sector 00. This pattern is noteworthy and suggests that drugs – particularly broadly acting classes such as antineoplastic and immunomodulating agents – can exert consistent and biologically meaningful effects on these core genes without destabilizing their ubiquitous expression (Fig. 4, Supplementary Fig. 4).

Importantly, we did not observe a direct correlation between gene ubiquity and drug effect sizes. This may indicate that drugs tend to stabilize the expression of highly ubiquitous genes, or that different drugs exert opposing effects on the same genes, effectively canceling each other out and preserving their overall ubiquitous expression profile.

Gene set enrichment analyses further highlighted functional differences between sector 11 and sector 10 genes (Fig. 5). Sector 11 genes are predominantly involved in biochemical pathways (KEGG, Reactome, WikiPathways) and show stronger associations with Human Phenotype Ontology (HP) terms, underscoring their relevance to genetic variation and disease mechanisms. In contrast, sector 10 genes are more strongly enriched for transcription factor binding sites, suggesting tighter regulation by transcriptional feedback loops. The increased dependence on transcription factor activity may underlie their more dynamic and variable expression changes and could explain their reduced stability as reference genes. A striking example of this is *TBP*, which itself is a crucial general transcription factor that indirectly influences the expression of many other genes.

Overall, the data indicate that ubiquitous expression is generally associated with stringent regulatory control. Importantly, occasional expression changes under pharmacologically perturbed conditions do not contradict the concept of ubiquity but rather reflect adaptive regulation of genes essential for maintaining cellular function.

### Other applications

The Ubigen method, together with its interactive web interface, is applicable beyond the study of ubiquitous genes and drug-induced effects. In particular, it enables the identification of reference genes tailored to specific tissue and experimental contexts. One illustrative application example is cancer research, as demonstrated for the “Glioma (TCGA)” dataset. In addition, systematic quantification of gene expression stability offers promising opportunities in aging research. Aging is closely associated with a progressive decline in core cellular homeostasis, and age-related transcriptional changes predominantly affect genes that are otherwise ubiquitously expressed. In this context, the Ubigen score can support the identification of robust biomarkers of aging [23, 24]. 

The approach may also be extended to longevity research. Gene expression signatures of long-lived species correlate positively with age-related changes and are enriched for evolutionarily ancient genes that are consistently expressed across tissues. Furthermore, the Ubigen framework may aid in exploring strategies to improve lifespan and healthspan. In humans, the most effective life-extending interventions, at least those identified so far, often target less conserved metabolic components. It would be worthwhile to investigate whether such interventions are associated with lower gene ubiquity and whether targeting less ubiquitous, more organ-specific pathways yields greater benefits in humans [25]. 

To further refine the concept of ubiquity, additional stressors could be incorporated into the analysis, including genetic perturbations from knockout studies and environmental challenges such as hypoxia or heat stress. The scoring framework introduced here is flexible and readily accommodates such extensions. In the context of differential expression analyses, it may also be advantageous to relax fold-change thresholds for genes with high Ubigen scores. Because these genes are typically expressed at high and stable levels, modest but biologically relevant changes may otherwise be overlooked.

In conclusion, the interactive user interface presented here is a valuable resource for both improving experimental design and advancing biological insight.

### Limitations

Several limitations should be considered when interpreting Ubigen Score results. *Dataset biases*: GTEx and CMap differ in technology (RNA-seq vs. microarrays), sample composition, and cell context. GTEx is based on RNA sequencing and includes expression data for 55,242 genes [21], whereas CMap relies on microarray technology and covers 12,382 genes [12]. Differences in sensitivity, dynamic range, and gene coverage may introduce systematic biases. To address sample inequality, we use the intersection of analyzed genes and annotated genes as the custom background for all gene set enrichment analyses. Nevertheless, this approach may still yield artificially elevated enrichment scores due to mutual compensation effects or uniformly small expression changes across conditions. *Cell line-specific constraints*: CMap’s cancer cell lines lack the genetic heterogeneity of primary tissues, potentially masking expression variability or exaggerating drug robustness. Importantly, our analysis does not primarily focus on sector 01 in Fig. 3, which may contain genes that appear unnaturally stable due to cell line artifacts. Rather, our focus is on genes that are expected to be ubiquitously expressed yet may show pronounced drug-induced changes, particularly those in sectors 11 and 10 of Fig. 3. Nevertheless, conclusions regarding drug sensitivity of ubiquitous genes should be interpreted with caution, and further experimental validation is required. *Annotation and selection bias*: Ubiquitously expressed genes tend to be better studied, better annotated, and more reliably detected across platforms. This may inflate their apparent ubiquity.

*Interpretation of drug effects*: Observed expression changes cannot be assumed causal or mechanistically linked to drug action without targeted validation experiments. The Ubigen score based on “CMap” merely reflects how consistently a gene is expressed across drug-perturbed samples under the experimental settings used by CMap. It does not imply that individual drugs directly regulate specific genes or that observed transcriptional changes translate into predictable functional outcomes. *Generalizability to other contexts*: Environmental stressors, genetic variation, and disease states beyond the datasets used here may alter ubiquity estimates; thus extending the method to additional perturbations would improve robustness and general applicability.

## Conclusion

In the introduction to this manuscript, we deliberately transitioned from the term “housekeeping genes” to “ubiquitous genes” to better reflect insights derived from large-scale expression data. Using the Ubigen score, we identified a set of genes whose expression remains stable across physiological conditions and under the influence of most drugs, and which are closely associated with fundamental cellular functions. We propose that this subset aligns more closely with the original conceptual definition of housekeeping genes.

Our findings demonstrate the limitations of rigid, canonical lists of housekeeping genes and underscore the value of a quantitative, data-driven framework. Although high Ubigen scores identify genes with stable expression, experimental conditions can still modulate their transcription on a large scale. Consequently, the use of multiple reference genes remains best practice for robust normalization in gene expression studies.

## Methods

### Data acquisition and retrieval

The “GTEx (all)” and “GTEx (tissues)” datasets were derived from public data provided by the Genotype-Tissue Expression (GTEx) Portal (https://gtexportal.org*)*, version 8 (ref [21]). The “GTEx (all)” dataset is based on raw RNA-seq read counts and was normalized as described below. The “HPA (tissues)” dataset was obtained from the Human Protein Atlas [25] at https://www.proteinatlas.org which also publishes aggregated gene expression data.

For the “CMap” dataset, we used a curated dataset from Struckmann et al. 2021 (ref [13])., which provides absolute expression values and fold changes derived from batch-corrected Affymetrix CEL files from the Connectivity Map [12]. The “Glioma (TCGA)” dataset was created using the Genomic Data Commons (GDC) Data Portal (https://portal.gdc.cancer.gov*).* We included all 1,244 glioma cases with RNA-seq data that were available without access restrictions [19, 20]. 

### Normalization of input data

Expression data for the default dataset spanning tissues and conditions (“GTEx (all)”) were normalized from raw RNA-seq read counts using the GeTMM method, which supports both intra- and inter-sample comparisons of expression levels [26]. Normalization followed the authors’ recommended implementation based on the “edgeR” package [27]. Transcript sequence lengths required for this procedure were retrieved from Ensembl, version 107 (ref [28]). The same normalization workflow was applied for the “Glioma (TCGA)” dataset. We did not perform any further normalization on the “GTEx (tissues)” and the “HPA (tissues)” dataset, as both datasets were provided as aggregated, pre-normalized expression summaries.

Genes in the “CMap” dataset are identified by numerical Entrez Gene IDs. We used functionality from the R package “gprofiler2” to map these identifiers into the Ensembl namespace for our analyses [29]. GTEx, HPA and TCGA datasets already use Ensembl identifiers.

### Data analysis

All analyses were implemented using the R programming language [30]. The full analysis code is freely available online (Code Availability) and most aspects of the analysis are configurable through functions provided in our R package “ubigen”, or via the interactive web interface at https://ubigen.uni-rostock.de.

The final scoring is based on a single data point per gene and per sample. Genes are consistently identified by their Ensembl ID. In rare cases, where duplicate entries or ambiguous mappings occurred, one gene entry was selected at random. The definition of a “sample” varies depending on the input dataset: For “GTEx (all)”, this directly corresponds to a physical sample within GTEx. For “GTEx (tissues)” and “HPA (tissues)”, a “sample” is actually a tissue, i.e. aggregated from multiple physical samples. For “CMap”, a “sample” refers to a combination of a single drug with a single concentration. The one data point for each combination of gene and sample is still called “expression level” for simplicity although it is actually the average expression level for “GTEx (tissues)”, “HPA (tissues)” and “CMap” and includes normalization steps as described above.

Threshold-based criteria (“Above 95th percentile”, “Above median” and “Above zero”) were calculated as the proportion of samples in which gene expression exceeded the selected threshold. For all other criteria, the named statistical values were computed across all samples, excluding samples in which the expression level is zero (“Median”, “Mean”, “Quartile-based coefficient of variation”, “Interquartile range”, “Coefficient of variation” and “Standard deviation”). This separation ensures independence between threshold-based and variability-based criteria. The range of values for the threshold-based criteria is already between 0.0 and 1.0. The other subscores are normalized to the [0.0, 1.0] range using min-max normalization. This means that a score of 1.0 corresponds to the highest observed value in the respective metric.

Final gene scores, ranks and percentiles were computed as a weighted average of three selected subscores capturing expression prevalence, overall expression level and expression variability (Table 1). Scores were normalized to the range [0.0, 1.0] to ensure comparability across different parameter configurations and weightings.

### Comparison datasets

Previously published lists of ubiquitously expressed or housekeeping genes were compiled through a manually curated literature search for the terms “housekeeping genes” and “ubiquitously expressed genes” on PubMed. Only studies providing gene lists as supplementary data could be included. All referenced studies are cited in the main text. Because these studies used heterogeneous gene identifier systems, all identifiers were mapped to Ensembl gene IDs using g: Convert from g: Profiler [18].

### Gene set enrichment analysis

All gene set enrichment analyses were performed using the tool “g: Profiler”^18^ and its R client package “gprofiler2” with the recommended parameter configuration [29]. The analyses are restricted to the intersection of annotated genes and genes included in the result dataset as a user-defined statistical background, which is recommended by the authors for situations where only part of the genome is studied due to technological or biological reasons [29]. It minimizes the bias introduced by the fact that some genes contained in different databases and datasets are more frequently studied and are likely to have more annotations. The corresponding visualizations are based on a custom re-implementation of the package’s plotting code. Sliding window gene set enrichment analyses were implemented in R as follows: The ranked gene data are divided into equally sized buckets containing the specified number of genes. The last bucket, containing the lowest ranking genes, may contain a smaller number of genes if the total number of genes is not divisible by the bucket size. A gene set enrichment analysis is performed separately for each bucket. For each bucket, the total number of significantly overrepresented terms is counted. This is based on our use of this method in Projahn et al. 2024 (ref [14]).

### Significance thresholds

The influence of individual drugs on individual genes is assessed using a t-test comparing treated and untreated samples, as provided by Struckmann et al. 2021 (ref [13]). We consider a single drug to significantly influence a single gene’s expression at *P* ≤ 0.05. All results that show effect sizes for groups of drugs (e.g., mechanisms of action or ATC classes) are descriptive summaries computed from gene-drug pairs meeting this criterion and are aggregated across drugs using the mean effect size (log fold change). We do not report P-values for these aggregate measures. For gene set enrichment analyses, multiple testing is corrected using the g: SCS method implemented in g: Profiler [18] with an adjusted significance threshold of P_adj_ ≤ 0.05.

### Visualizations and graphical user interface

All figures presented in this study and on the interactive web interface were generated using the “plotly” R package. The web interface was implemented using the “Shiny” framework. All code related to visualization and the web application is freely available online (see Code Availability) [31].

## Supplementary Information

Supplementary Material 1

Supplementary Material 2

Supplementary Material 3

Supplementary Material 4

Supplementary Material 5

Supplementary Material 6

Supplementary Material 7

Supplementary Material 8

Supplementary Material 9

## Data Availability

The interactive web interface developed for this study is publicly accessible at https://ubigen.uni-rostock.de. The website provides access to all default Ubigen scores, which can be downloaded as a complete dataset and are also included as Supplementary Table 6. Supplementary Table 4 contains detailed information for all genes classified as ubiquitous based on their assignment to sector 11 in Figure 3, Supplementary Table 5 provides the full list of analyzed genes together with their corresponding sector assignments.
