# Supplementary material for: Multicondition expression profiling reveals limitations of canonical housekeeping genes
Source: BMC Genomics. 2026 Jan 27;27:214. doi: 10.1186/s12864-026-12563-8 (PMC12918141; doi:10.1186/s12864-026-12563-8)
Supplement: Supplementary file 1 — Supplementary Material 1 [file 12864_2026_12563_MOESM1_ESM.pdf]

Supplements

Supplementary Table 1: Correlation between Ubigen scores for different datasets.

	GTEx (all)	GTEx (tissues)	HPA (tissues)	CMap
**GTEx (all)**	1.0000	0.8008	0.5612	0.4475
**GTEx (tissues)**	0.8008	1.0000	0.6646	0.5334
**HPA (tissues)**	0.5612	0.6646	1.0000	0.5261
**CMap**	0.4475	0.5334	0.5261	1.0000

This table presents the correlation matrix of Ubigen scores across multiple datasets. The reported values are Spearman's rank correlation coefficients (ρ) calculated using genes shared between each pair of datasets. Strong correlations are observed among "GTEx (all)", "GTEx (tissues)" and "HPA (tissues)" ($0.5612 \leq \rho \leq 0.8008$) while "CMap" shows weaker correlations with the other datasets ($0.4475 \leq \rho \leq 0.5334$).

Supplementary Table 2: Detailed results of the gene set enrichment analysis for the top 2.5% of genes in the ranking based on "GTEx (all)". The analysis was performed using g:Profiler.

Supplementary Table 3: Genes common to previously published datasets of ubiquitous genes. These 88 genes correspond to the intersection of the five datasets shown in Supplementary Figure 1. Results based on "GTEx (all)" and "CMap" are included.

Supplementary Table 4: Newly reported ubiquitous genes. This table contains Ubigen scores for genes in sector 11 of Figure 3. These are the genes that are ubiquitous under physiological circumstances and that are also ubiquitous when considering drug effects.

Supplementary Table 5: Sectors for all genes. This table contains Ubigen scores for all genes and their resulting sectors based on Figure 3.

Supplementary Table 6: Ubigen scores for all available genes. This data is based on all samples from the GTEx project. Scores and ranks are calculated using the default criteria and weights described in Table 1.

Supplementary Table 7: Scores based on tissue-aggregated data from GTEx. Scores and ranks are calculated using the default criteria and weights described in Table 1.

Supplementary Table 8: Scores based on tissue-aggregated data from HPA. Scores and ranks are calculated using the default criteria and weights described in Table 1.

Supplementary Table 9: Scores based on CMap. Scores and ranks are calculated using the default criteria and weights described in Table 1.

Supplementary Figure 1: Venn diagram illustrating the overlap between previously published sets of housekeeping or ubiquitously expressed genes.

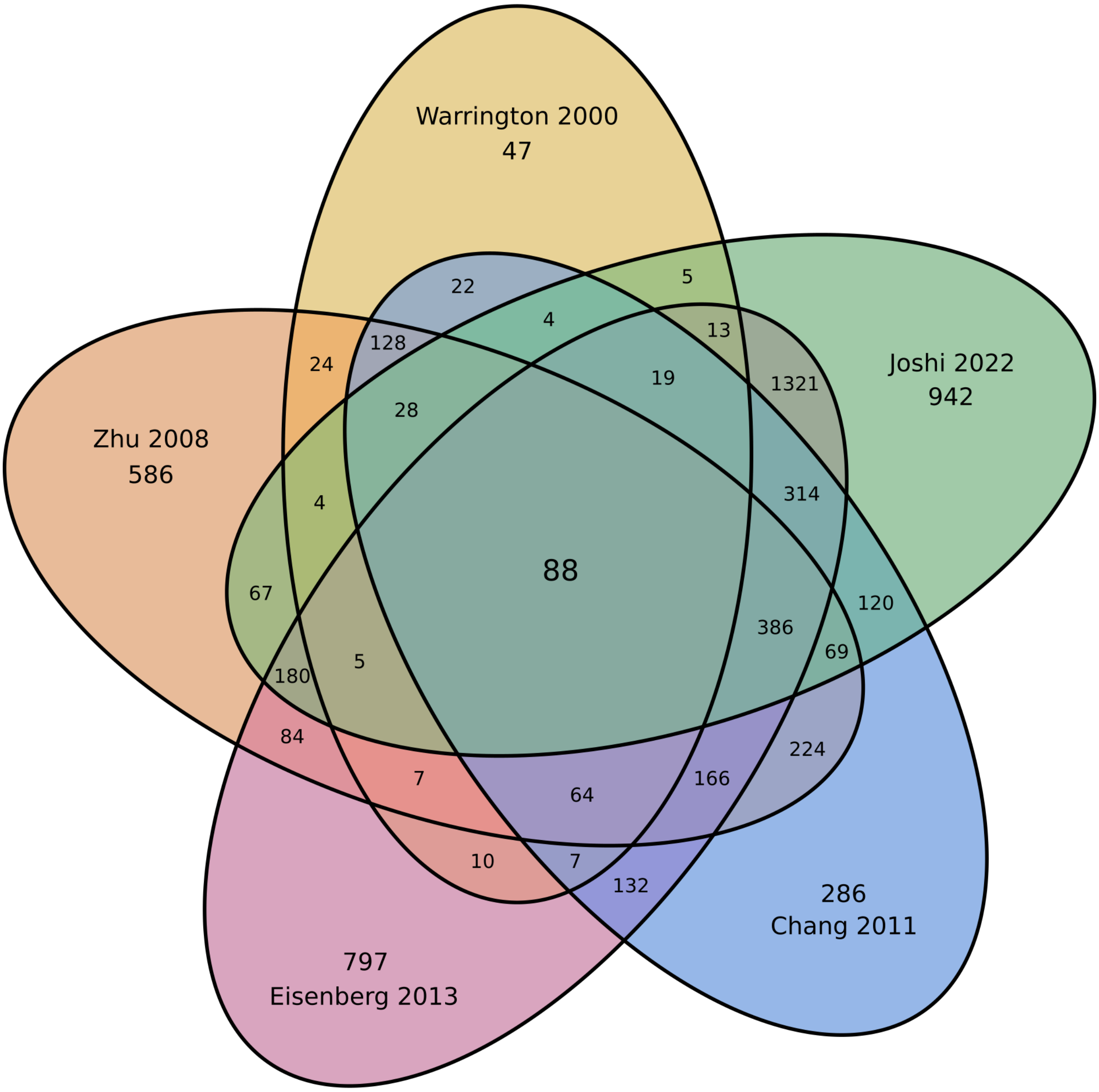

This diagram depicts the intersections among gene sets that have been reported in the literature as housekeeping or ubiquitously expressed genes based on expression data. Only 88 genes are shared across all five datasets. These 88 genes were compiled into an additional comparison dataset, referred to as "Venn 88".

Supplementary Figure 2: Distribution of commonly used datasets of ubiquitously expressed genes across Ubigen sectors.

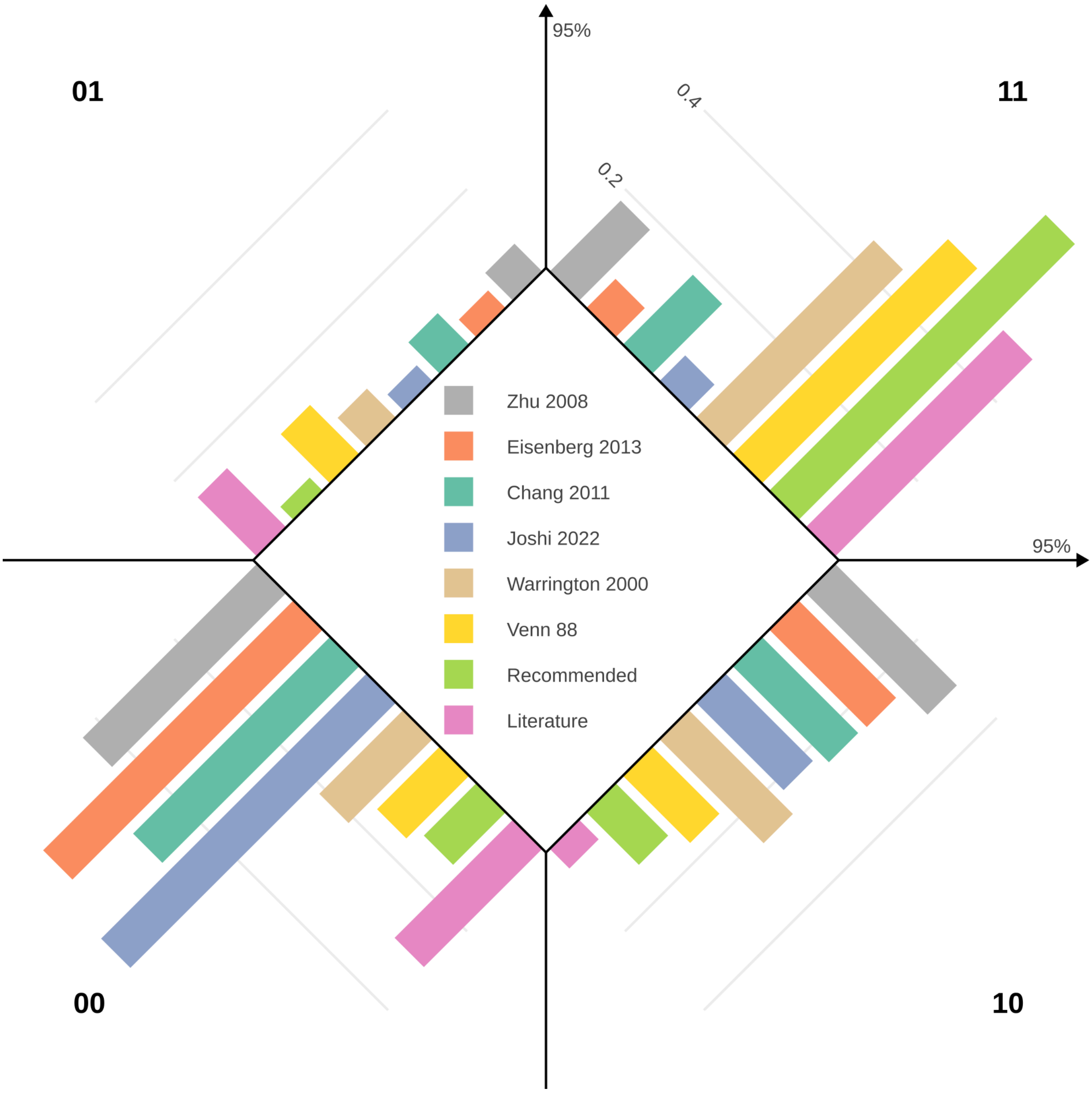

This figure illustrates how widely cited datasets of ubiquitously expressed or housekeeping genes (Zhu 2008, Eisenberg 2013, Chang 2011, Joshi 2022, Warrington 2000) are distributed across the sectors defined in Figure 3. With the exception of Warrington (2000), only a small fraction of genes in these datasets meet the criteria for ubiquity when drug-induced expression changes are taken into account. This observation is only partly explained by reduced specificity (i.e., assignment to sector 00 rather than sector 11), as genes in sector 10 still substantially outnumber those in sector 11. The "Venn 88" dataset represents the intersection of all five datasets and shows improved performance compared with individual datasets. The "Recommended" dataset includes genes explicitly recommended by the respective publications based on human-focused considerations, while the "Literature" dataset comprises commonly used reference summarized in Table 3. In these three smaller, more stringently curated datasets, an absolute majority of genes are assigned to sector 11, indicating greater robustness and consistency under the Ubigen framework.

Supplementary Figure 3: Drug scores derived from their effects on gene expression.

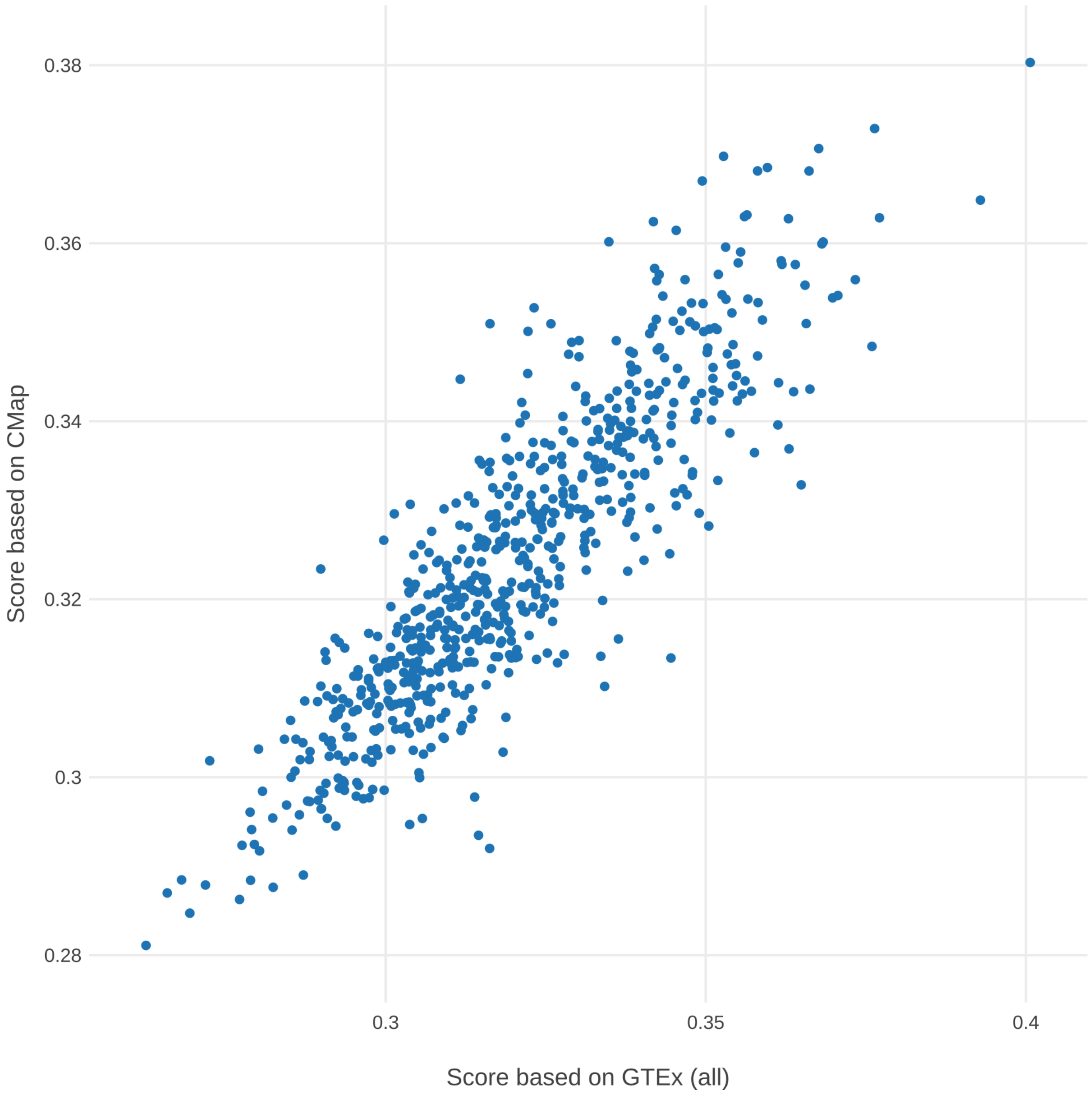

This figure presents scores for individual drugs based on the genes whose expression they significantly influence. For each drug, a composite score is calculated by averaging the Ubigen scores of all affected genes derived from the “GTEx (all)” dataset (x-axis) and the “CMap” dataset (y-axis), with weights proportional to the magnitude of the corresponding fold-changes. Consequently, the position of each drug in the plot reflects the overall ubiquity of the genes it modulates. An interactive version of this figure, including drug names and additional metadata, is available through the web interface in the “Additional information” section.

Supplementary Figure 4: Drug-induced effects across groups of ubiquitous genes.

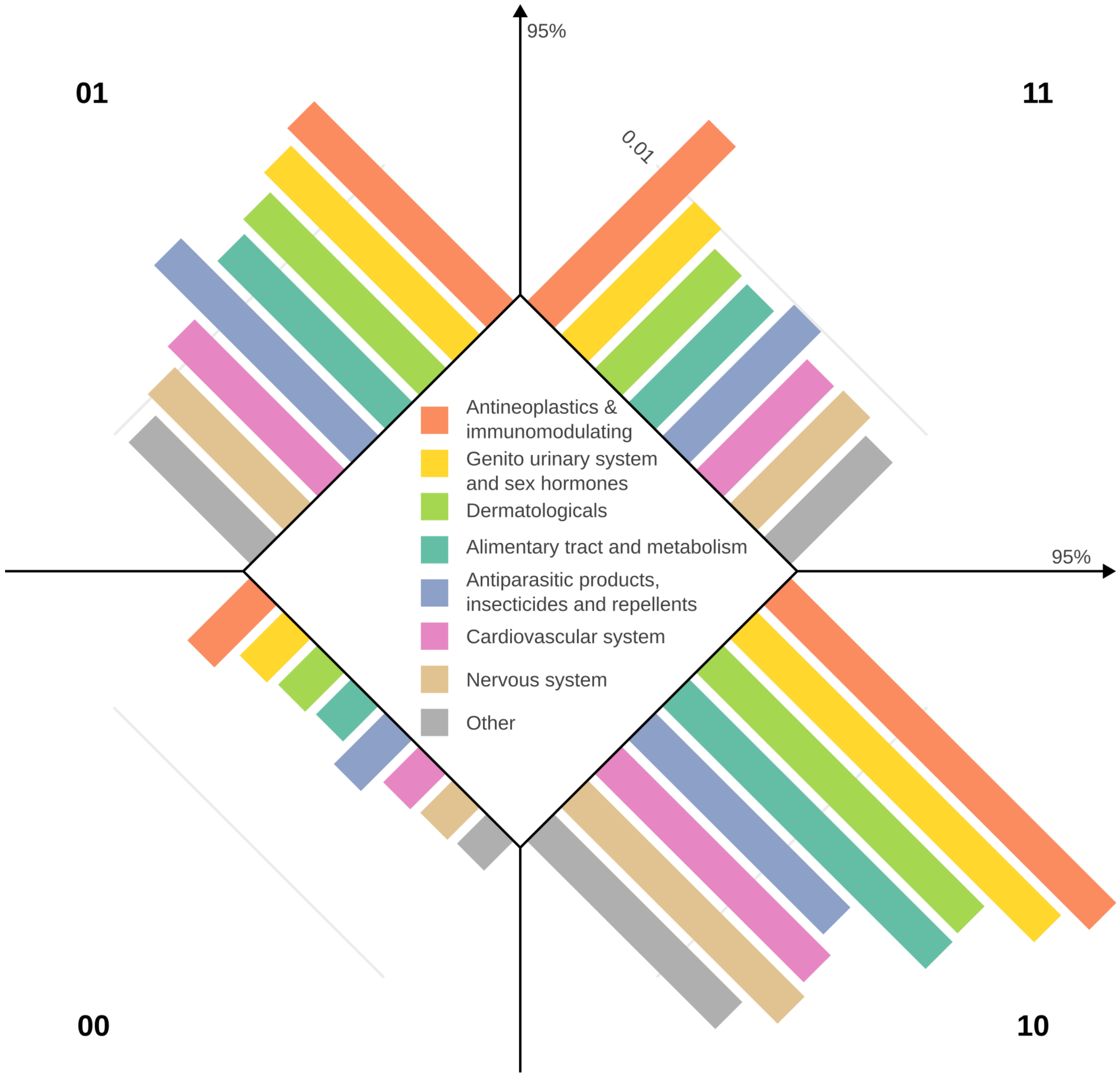

This figure illustrates differences in drug-induced expression changes across the gene sectors defined in Figure 3. For each sector, the effects of drugs grouped by World Health Organization Anatomical Therapeutic Chemical (WHO ATC) level 1 categories are shown. Effect sizes are calculated as the average log fold change in gene expression for genes within a given sector, aggregated across all drugs belonging to the respective category. Overall, the largest effect-sizes are observed for genes that are ubiquitously expressed under physiological conditions but are responsive to drug perturbation (sector 10), while non-ubiquitous genes (sector 00) exhibit substantially smaller effects. Antineoplastic and immunomodulating agents (ATC code L) show the strongest effects across all sectors and are particularly effective at modulating genes that remain ubiquitous even when drug effects are considered (sector 11).
